# Draft genome sequences data of two Rosemountvirus phages isolated from soil near poultry farm

**DOI:** 10.1016/j.dib.2022.108488

**Published:** 2022-07-23

**Authors:** A. Bogoyavlenskiy, M. Alexyuk, P. Alexyuk, Y. Moldakhanov, V. Berezin

**Affiliations:** Research and Production Center for Microbiology and Virology, Almaty, Kazakhstan

**Keywords:** Bacteriophage, Genome, Myoviridae, Escherichia coli

## Abstract

Here we report the draft complete genome sequence of Escherichia phage vB_EcoM_IntR and Escherichia phage vB_EcoM_PiR isolated from a poultry farm soil sample. Bacteriophage isolation on the model of the pathogenic for birds *Escherichia coli* was carried out. Sequencing was carried out on the Illumina platform. Phage genomes were assembled using a software Geneious package. Phage sequences do not contain tRNA. Phages are a linear double-stranded DNA from Rosemountvirus genus of Myoviridae family with a genome 52,782 and 52,936 bp, respectively, containing 71 predicted open reading frames (ORFs). The phages have a standard icosahedral head and a contractive tail characteristic of the myovirus family.

## Specifications Table

**Table:** 

Subject	Biology
Specific subject area	Bacteriophages
Type of data	Whole genome sequence data
How the data were acquired	Next generation sequencing on Illumina platform
Data format	Raw, analysed
Description of data collection	Genome sequence of phages vB_EcoM_IntR and vB_EcoM_PiR
Data source location	Research and Production Center for Microbiology and Virology, Almaty, Kazakhstan (N 43° 42′ 52.992" E 77° 2′ 27.996").
Data accessibility	https://data.mendeley.com/datasets/hkd89s56kv/1https://www.ncbi.nlm.nih.gov/nuccore/OM313460https://www.ncbi.nlm.nih.gov/nuccore/OM313461
Related research article	none

## Value of the Data

• Study of the genomes of new lytic phages with a view to their possible application against Avian Pathogenic *Escherichia coli.*
• Data provides information on phage genes and proteins encoded by those genes.
• Data may assist researchers in sequence comparison.
• Data can be used by researchers for the genomic, proteomic and other of evolutionary studies
• Data ensures no toxic gene carried by phage, which makes it a potent agent for applications

## 1 Data Description

**Fig. 1:**
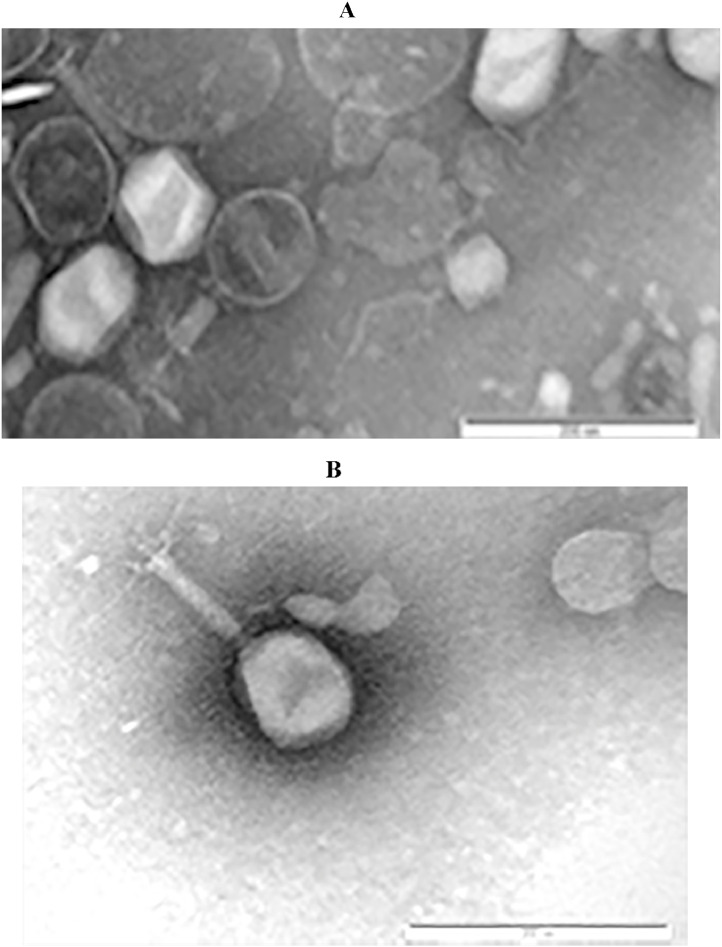
Phage morphology by transmission electron microscopy, A. Escherichia phage vB_EcoM_IntR, B. Escherichia phage vB_EcoM_PiR.

**Fig. 2:**
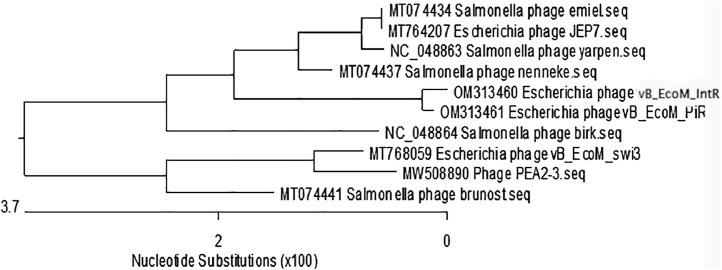
Phylogenetic tree of Rosemountvirus, Myoviridae on the model of the tape measure protein.

Phages were isolated from soil sample near poultry farm at geographical location (N 43°42′ 52.992" E 77°2′27.996”). They were isolated on the model of the bacterium *E. coli*, which is pathogenic for birds. Transmission electron microscopy showed typical morphology characteristic of myoviruses (Fig. 1). The tape measure protein was used for investigation the evolutionary patterns and founding that viruses form a separate monophyletic group among members of the genus Rosemountvirus of Myoviridae family (Fig. 2). The Table 1 shows brief characteristics of the genome of the studied viruses. The average size of the forward and reverse reads after trimming was 285 bp. The number of reads during sequencing on the Illumina platform was 259889 and 310252, respectively.

**Table 1:** Genome sequence characteristics of phage.

Index	Value vB_EcoM_IntR	Value vB_EcoM_PiR
Genome size	52,782 bp	52,936 bp
GC content	45,9 %	45,8 %
ORF	71	71
Accession no.	OM313460	OM313461

## 2 Experimental Design, Materials and Methods

For transmission electron microscopy 30 microliters of each purified phage lysate was spotted on formvar coated copper grid (300 mesh) for 1 min. Then, 3% phosphotungstic acid (ph 6,8) was used to stain the samples, and excess solution was removed. The grids were allowed to air dry and were then observed with a JEM-2100 “JEOL” (Japan) transmission electron microscope.

**Fig. 3:**
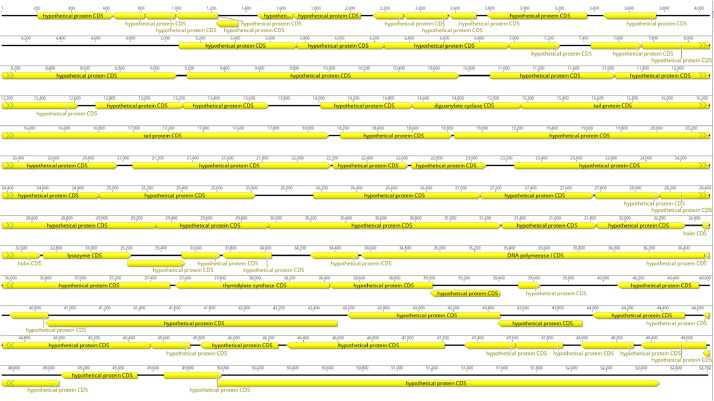
Whole genome map of Escherichia phage vB_EcoM_IntR.

**Fig. 4:**
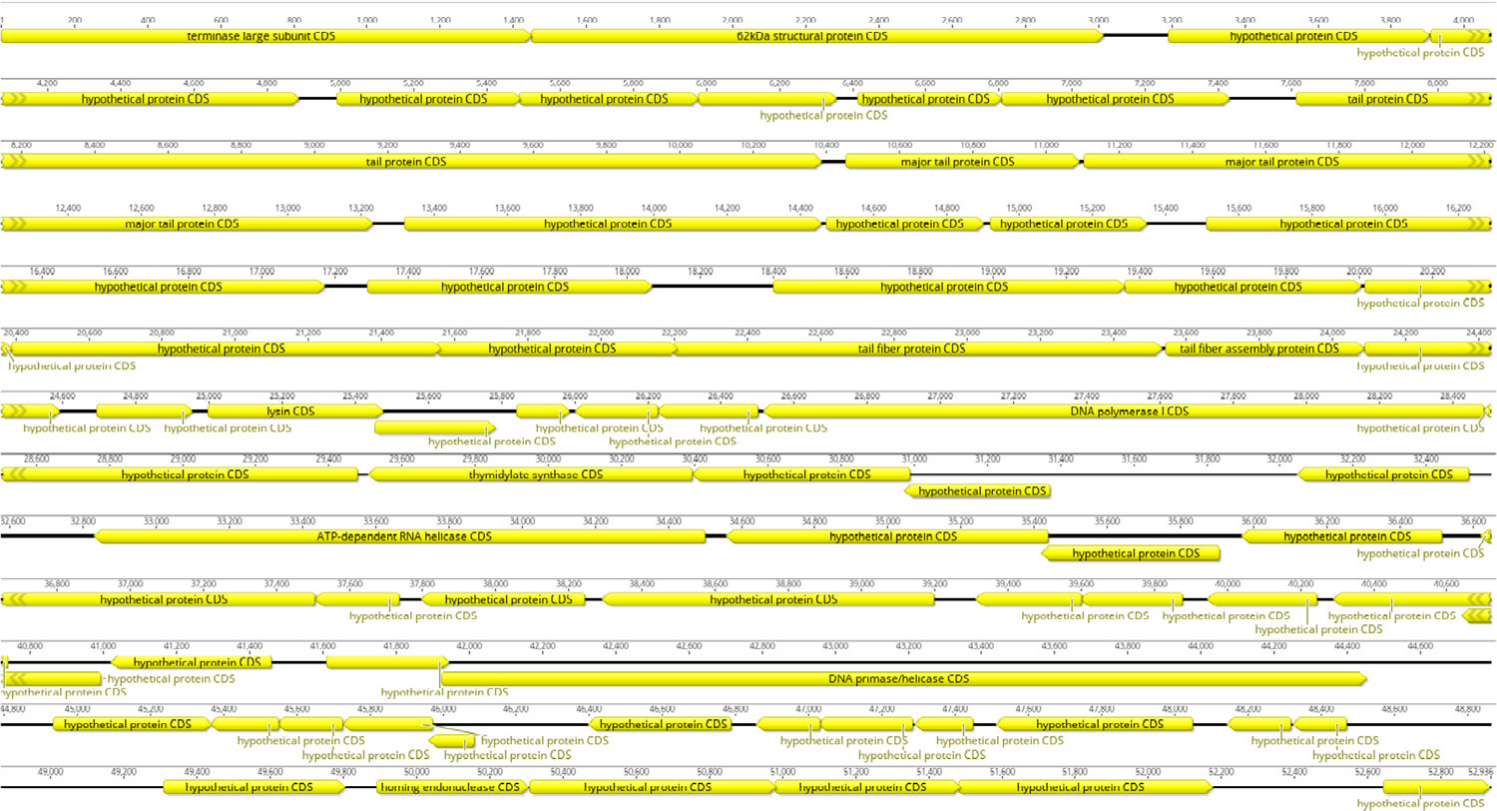
Whole genome map of Escherichia phage vB_EcoM_PiR.

The genomic DNA from the phage lysate of the *E. coli* isolate was extracted according to the guide for the PureLink viral DNA/RNA minikit (Thermo Fisher Scientific, USA). The DNA library was prepared using the Nextera XT DNA sample preparation kit (Illumina). Whole-genome sequencing was performed with an Illumina MiSeq sequencing platform. Low-quality reads were filtered and adapters were trimmed with Trimmomatic [1] from the Genome Detective tool [2]. As a result, a total of paired-end reads were assembled using SPAdes 3.12.0 [3]. The average read length after trimming was 286 bp. The genomes were assembled to an average depth of more than 1,000 [4,5]. The physical ends of the viral genome were identified by comparing the coverage values of the complete genome length of the virus Escherichia phage vB_EcoM_IntR and the closely related viruses Escherichia phage vB_EcoM_PiR in the NCBI database with BLAST [6], namely, Escherichia phage vB_EcoM_swi3 (MT768059.1) with 100% query coverage and 95.93% identity and Escherichia phage JEP7 (MT764207.1), with 100% query coverage and 97.07% identity. Phages vB_EcoM_IntR and vB_EcoM_PiR are composed of double-stranded DNA that are 52782 and 52936 bp in length, respectively and belongs to Rosemountvirus genus, Myoviridae. The GC content of the genomes was 45.9% and 45.8% respectively. Gene prediction was done using GeneMark and PHAST [7,8]. A total of 71 putative genes were predicted in the complete genome of both phages (Fig. 3, 4).

**Nucleotide sequence accession numbers:** OM313460 and OM313461

## Ethics Statements

Work did not include human subjects, animal experiments or data collected from social media platforms

## CRediT Author Statement

**A. Bogoyavlenskiy:** Conceptualization, Methodology, Software, Writing – original draft preparation; **M. Alexyuk:** Data curation, Writing – review & editing; **P. Alexyuk:** Software, Validation; **Y. Moldakhanov:** Investigation; **V. Berezin:** Supervision.

## Declaration of Competing Interest

The authors declare that they have no known competing financial interests or personal relationships that could have appeared to influence the work reported in this paper.

## Data Availability

raw sequence data (Original data) (Mendeley Data).

## References

[1] Bolger A.M., Lohse M., Usadel B. (2014). Trimmomatic: a flexible trimmer for Illumina sequence data. Bioinformatics.

[2] Vilsker M., Moosa Y., Nooij S., Fonseca V., Ghysens Y., Dumon K., Pauwels R., Alcantara C.L., Eynden E.V., Vandamme A.M., Deforche K., de Oliveira T. (2019). Genome Detective: an automated system for virus identification from high-throughput sequencing data. Bioinformatics.

[3] Bankevich A., Nurk S., VAntipov D., Gurevich A., Dvorkin M., Kulikov A., Lesin V., Nikolenko S., Pham S., Prjibelski A., Pyshkin A., Sirotkin A., Vyahhi N., Tesler G., Alekseyev M., Pevzner P. (2012). SPAdes: a new genome assembly algorithm and its applications to single-cell sequencing. J. Comput. Biol..

[4] Kearse M., Moir R., Wilson A., Stones-Havas S., Cheung M., Sturrock S., Buxton S., Cooper A., Markowitz S., Duran C., Thierer T., Ashton B., Meintjes P., Drummond A. (2012). Geneious basic: an integrated and extendable desktop software platform for the organization and analysis of sequence data. Bioinformatics.

[5] Altschul S.F., Gish W., Miller W., Myers E.W., Lipman D.J. (1990). Basic local alignment search tool. J. Mol. Biol..

[6] Deng W., Nickle D.C., Learn G.H., Maust B., Mullins J.I. (2007). ViroBLAST: a stand-alone BLAST Web server for flexible queries of multiple databases and user's datasets. Bioinformatics.

[7] Besemer J., Borodovsky M. (2005). GeneMark: Web software for gene finding in prokaryotes, eukaryotes and viruses. Nucl. Acids Res.

[8] Zhou Y., Liang Y., Lynch K.H., Dennis J.J., Wishart D.S. (2011). PHAST: a fast phage search tool. Nucl. Acids Res.

